# Therapeutic Effects of *Akebia quinata* Seeds Through Apoptosis and Immunogenic Cell Death in Non-Small Cell Lung Cancer

**DOI:** 10.3390/ijms27073114

**Published:** 2026-03-30

**Authors:** Mibae Jeong, In Jin Ha, Chang-Seob Seo, Mi-Kyung Jeong, Kwang Seok Ahn, Jaemoo Chun

**Affiliations:** 1 KM Convergence Research Division, Korea Institute of Oriental Medicine, Daejeon 34054, Republic of Korea; 2 Department of Science in Korean Medicine, College of Korean Medicine, Kyung Hee University, Seoul 02447, Republic of Korea; 3 Korean Medicine Clinical Trial Center (K-CTC), Korean Medicine Hospital, Kyung Hee University, Seoul 02447, Republic of Korea; ijha@khu.ac.kr; 4 KM Science Research Division, Korea Institute of Oriental Medicine, Daejeon 34054, Republic of Korea; csseo0914@kiom.re.kr; 5 KIOM School, University of Science & Technology (UST), Daejeon 34054, Republic of Korea

**Keywords:** *Akebia quinata* seeds, saponin, non-small cell lung cancer, apoptosis, immunogenic cell death, mitogen-activated protein kinase

## Abstract

Plant-derived saponins have attracted significant interest for their potential to promote apoptotic cell death and enhance antitumor immune responses through immunogenic cell death (ICD). *Akebia quinata*, a saponin-rich medicinal plant, exhibits diverse pharmacological properties; however, studies on its seeds are limited, and their immunomodulatory activity in cancer remains largely unexplored. In this study, *A. quinata* seeds were extracted using 70% ethanol, and the phytochemical profile was characterized using UHPLC–QTOF MS/MS. We investigated the anticancer properties of *A. quinata* seed extract (AQSE), focusing on its role in inducing apoptosis and ICD in non-small cell lung cancer (NSCLC). In human NSCLC cell lines (A549 and H460), AQSE exhibited potent cytotoxic effects in a dose-dependent manner. Flow cytometric analysis confirmed the induction of apoptosis, evidenced by a significant increase in Annexin V-positive cells and an elevated sub-G1 population. Mechanistically, AQSE treatment induced cell death by simultaneously inhibiting the survival-promoting MEK/ERK/CREB axis and activating the stress-responsive JNK pathway. Furthermore, AQSE triggered hallmark features of ICD, characterized by surface exposure of calreticulin and the release of extracellular HMGB1 and ATP. Most importantly, an in vivo vaccination assay using a syngeneic mouse model demonstrated that immunization with AQSE-treated dying cells significantly suppressed tumor growth upon rechallenge, confirming the establishment of antitumor immunological memory. Additionally, bioassay-guided fractionation revealed that the anticancer activity was primarily concentrated in the ethyl acetate fraction. These findings suggest that AQSE exerts anticancer effects via the induction of apoptosis and ICD, highlighting its potential as a promising natural candidate for the development of novel therapeutic strategies against NSCLC.

## 1. Introduction

Lung cancer remains a major global health burden and continues to be the leading cause of cancer-related mortality worldwide, with non-small cell lung cancer (NSCLC) accounting for the majority of cases [1,2]. Despite significant advances in chemotherapy, radiotherapy, and targeted therapy, clinical outcomes for patients with advanced NSCLC remain limited. Immune checkpoint inhibitors (ICIs) have emerged as a promising therapeutic option and can induce durable responses in a subset of patients by restoring impaired antitumor immunity [3]. However, the majority of patients fail to respond or eventually develop resistance to ICIs, underscoring the need for complementary strategies that enhance tumor immunogenicity and improve immune-mediated tumor control. Accumulating evidence indicates that an immunosuppressive tumor microenvironment and the low immunogenicity of cancer cells represent key barriers to effective immunotherapy in NSCLC [4,5]. These immunologically silent features create a formidable obstacle, preventing the host immune system from effectively recognizing and eliminating tumor cells [6,7]. Consequently, there is an urgent need for complementary strategies that can remodel the tumor microenvironment and enhance tumor immunogenicity to overcome immunotherapy resistance.

Immunogenic cell death (ICD) has gained attention as a potential approach to increase the immunogenicity of poorly immunogenic tumors [8]. ICD is a distinct form of regulated cell death characterized by the release of damage-associated molecular patterns (DAMPs), including calreticulin (CALR) exposure on the cell surface, extracellular ATP secretion, and the release of high-mobility group box 1 (HMGB1) [9]. These signals are recognized by pattern recognition receptors on antigen-presenting cells, particularly dendritic cells, leading to their recruitment, maturation, and enhanced antigen presentation [10]. Consequently, ICD facilitates efficient cross-priming of cytotoxic CD8+ T lymphocytes and promotes antitumor immune responses. Importantly, ICD is increasingly recognized not merely as a form of cancer cell death, but as an immunological process capable of reshaping the tumor microenvironment and influencing therapeutic responsiveness in NSCLC [11,12].

Beyond direct cytotoxicity, ICD can confer immunogenic properties on dying tumor cells, promoting immune priming and long-term memory formation. Tumor cells undergoing ICD have been shown to function as a source of endogenous tumor vaccines, capable of eliciting protective antitumor immune responses [13]. Therefore, identifying agents that can trigger the release of key ICD biomarkers serves as a critical step in discovering new immunotherapeutic candidates. Given the emerging role of ICD in shaping antitumor immunity, the identification of novel and effective ICD-inducing agents remains an unmet need in NSCLC [14]. Although several conventional chemotherapeutic and targeted agents have been reported to mediate ICD under specific conditions, their clinical application is often limited by toxicity or insufficient immunogenic potency [15]. Therefore, the exploration of natural compounds that can induce ICD while simultaneously exerting anticancer activity represents a promising strategy to enhance immune-mediated tumor control in NSCLC.

Natural products represent a rich source of bioactive compounds with diverse pharmacological and immunomodulatory properties [16]. Among them, saponins have attracted increasing attention due to their membrane-active characteristics and their reported ability to influence inflammatory and immune responses [17]. Notably, specific saponins possess immunostimulatory properties similar to vaccine adjuvants. For instance, QS-21, derived from *Quillaja saponaria*, is a clinically established adjuvant used to potentiate antigen-specific T cell responses [18]. Similarly, saponins such as ginsenosides and platycodin D have been reported to exert adjuvant activity by inducing the secretion of inflammatory cytokines and the recruitment of immune cells, reinforcing the rationale for exploring saponin-based strategies to elicit protective antitumor immunity [19,20,21]. *Akebia quinata*, a medicinal plant belonging to the Lardizabalaceae family, has long been used in East Asian traditional medicine and is known to contain abundant triterpenoid saponins [22]. Previous studies have reported various biological activities of *A. quinata*, including anti-inflammatory, hepatoprotective, and metabolic regulatory effects [23,24,25]. However, studies specifically targeting *A. quinata* seeds (AQS) remain limited, and their phytochemical constituents have not been fully characterized. Furthermore, the anticancer mechanisms of *A. quinata*, particularly with respect to cancer cell death and tumor immunogenicity, remain largely unexplored [26]. In particular, whether AQS can induce apoptosis-associated stress responses and promote ICD has not been elucidated.

In this study, we investigated the anticancer effects of AQS in NSCLC cells, with a focus on their ability to induce apoptosis and ICD. We also explored the underlying molecular mechanisms, specifically the involvement of mitogen-activated protein kinase (MAPK) signaling pathways, which play a pivotal role in regulating cellular stress responses and cell death [27]. Furthermore, we examined whether AQS-treated tumor cells acquire vaccine-like properties in vivo. Through this integrative approach, we aimed to determine whether AQS could serve as a potential therapeutic candidate that enhances both cancer cell death and antitumor immunity in NSCLC.

## 2. Results

### 2.1. Identification of Chemical Components in A. quinata Seed Extract (AQSE)

**Figure 1:**
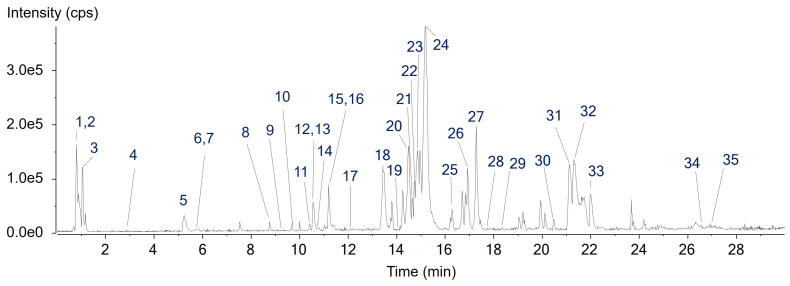
The representative base peak chromatogram of *A. quinata* seed extract (AQSE) in negative ion mode.

The chemical composition of AQSE was profiled using ultrahigh-performance liquid chromatography combined with quadrupole time-of-flight mass spectrometry (UHPLC–QTOF MS). Chromatographic profiles were assessed by examining total ion chromatograms and base peak chromatograms to obtain an overview of the chemical features of AQSE and to identify prominent peaks across the retention time range (Figure 1). For compound annotation, mass spectral data corresponding to major chromatographic peaks were further analyzed. Molecular formulae were assigned based on high-resolution mass measurements and isotopic distribution patterns using PeakView 2.2 and MasterView software 1.1 (SCIEX, Foster City, CA, USA). Putative identification of the major constituents was performed by comparing retention times, exact masses, and MS/MS fragmentation patterns with reference standards, in-house MS/MS libraries, and publicly available databases, including GNPS, MassBank, and METLIN. Based on these analyses, 35 compounds were identified as the constituents of AQSE). These included phenylpropanoids (C6–C3), flavonoids, triterpenoids, and fatty acids. Comprehensive chromatographic and mass spectrometric parameters were listed in Table 1.

### 2.2. AQSE Reduced Cell Viability in NSCLC Cells

**Figure 2:**
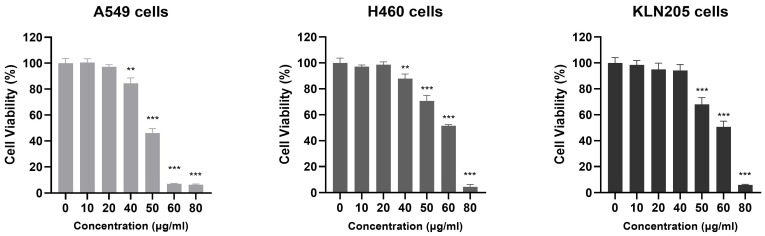
AQSE potently inhibits the growth of NSCLC cells. A549, H460, and KLN205 cells were treated with the indicated concentrations (0–80 μg/mL) of AQSE for 24 h. Cell viability was determined using the Cell Counting Kit-8 (CCK-8) assay. Data are presented as mean ± SD. ** *p* < 0.01 and *** *p* < 0.001, compared to the control group (0 μg/mL).

The effect of AQSE on cell viability was evaluated in A549, H460, and KLN205 cancer cells following treatment with increasing concentrations (0, 10, 20, 40, 50, 60, and 80 μg/mL) of AQSE for 24 h. AQSE treatment resulted in a significant reduction in cell viability across all tested cell lines (Figure 2). While lower concentrations (10–20 μg/mL) did not induce marked changes compared to the untreated control, higher concentrations (40–80 μg/mL) led to a pronounced decrease in viability. Notably, significant cytotoxicity was observed starting at 50 μg/mL for A549 cells, and at 60 μg/mL for H460 and KLN205 cells. Although the overall dose-responsive pattern was consistent across the three cell lines, the extent of the decrease varied, indicating differential sensitivities to the AQSE treatment. These results demonstrate that AQSE exerts potent cytotoxic effects against NSCLC cells.

### 2.3. AQSE Induced Apoptosis in NSCLC Cells

**Figure 3:**
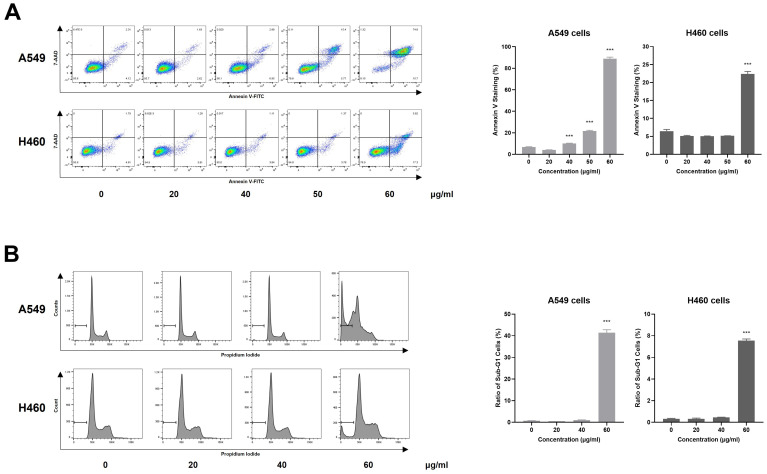
AQSE sensitizes A549 and H460 cells to apoptosis. (**A**) Flow cytometric analysis of apoptotic cells detected by Annexin V/7-AAD staining. Cells were treated with the indicated concentrations (0, 20, 40, 50, and 60 µg/mL) of AQSE for 24 h. Cells were stained with Annexin V-FITC and 7-AAD. The percentage of Annexin V-positive cells was quantified. (**B**) Cell cycle analysis of AQSE-treated cells. Cells were treated with the indicated concentrations (0, 20, 40, and 60 µg/mL) of AQSE for 24 h and stained with propidium iodide to determine DNA content. The histograms show the distribution of cell cycle phases, with bars indicating the sub-G1 population. Data are presented as mean ± SD. *** *p* < 0.001, compared to the control group (0 μg/mL).

To investigate whether the cytotoxic effects of AQSE were mediated by apoptosis, Annexin V/7-AAD staining and cell cycle analyses were performed on A549 and H460 cells. Cells were exposed to increasing concentrations of AQSE for 24 h and subsequently analyzed by flow cytometry. AQSE treatment increased the population of Annexin V/7-AAD double-positive cells in both cell lines, indicating progression toward late apoptosis (Figure 3A). Quantitative analysis further confirmed a significant increase in the percentage of Annexin V-positive cells following AQSE treatment. A549 cells exhibited a significant elevation in apoptosis at 50 and 60 μg/mL, whereas H460 cells showed marked induction only at 60 μg/mL. These findings indicate differential sensitivity to AQSE between the two cell lines, while confirming a consistent pro-apoptotic effect at higher doses. Consistent with these findings, cell cycle analysis revealed a marked accumulation of cells in the sub-G1 phase upon AQSE exposure (Figure 3B). Lower concentrations of AQSE (20 and 40 µg/mL) resulted in minimal alterations in cell cycle profiles in both A549 and H460 cells. In contrast, exposure to 60 µg/mL AQSE led to a substantial accumulation of cells in the sub-G1 phase, a hallmark of apoptotic DNA fragmentation. Quantification of sub-G1 populations confirmed a significant increase at the highest concentration tested in both cell lines.

### 2.4. AQSE Regulates Apoptosis-Related Proteins and Suppresses Survival Signaling Pathways

**Figure 4:**
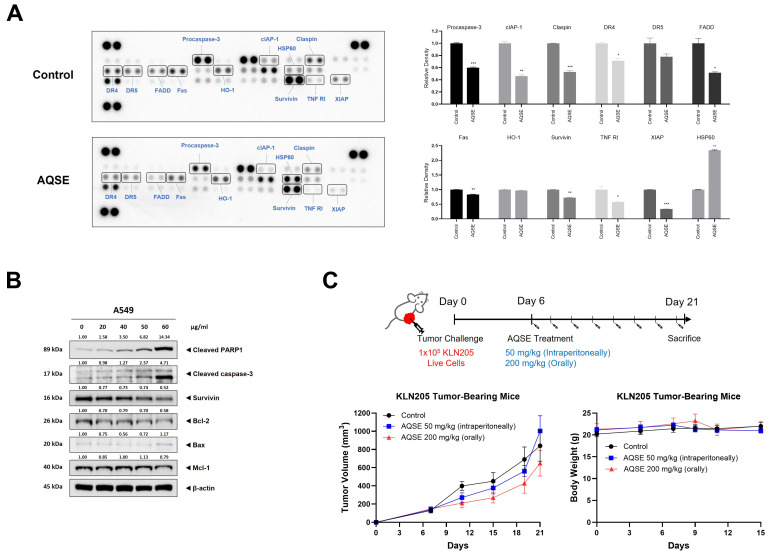
AQSE regulates apoptosis-related signaling. (**A**) Protein array showing the expression of apoptosis-related proteins in control and AQSE-treated A549 cells. Cells were treated with 50 μg/mL AQSE for 24 h. A quantitative comparison of selected protein levels is shown as relative density compared to the control. Data are presented as mean ± SD. * *p* < 0.05, ** *p* < 0.01, and *** *p* < 0.001, compared to the untreated control. (**B**) Western blot analysis confirming the expression levels of apoptosis-related proteins in A549 cells. (**C**) Changes in tumor volume and body weight in KLN205-bearing mice. KLN205 cells were inoculated subcutaneously into the right flank of mice. One week after injection, mice were administered AQSE intraperitoneally (50 mg/kg) or orally (200 mg/kg) every other day. Tumor volumes were measured every 2–4 days. Data are presented as mean ± SEM for tumor volume and mean ± SD for body weight.

To explore the molecular events associated with AQSE-induced cell death, an apoptosis-related protein array was performed in A549 cells. As shown in Figure 4A, AQSE treatment (50 μg/mL) markedly downregulated the expression of key anti-apoptotic proteins, including procaspase-3, cIAP-1, Claspin, Survivin, TNF RI, and XIAP, compared to the control group. Furthermore, death receptor-associated factors (DR4, DR5, FADD, and Fas) were also decreased, while the stress response-related proteins HSP60 was increased. To validate these findings, we performed Western blot analysis (Figure 4B). Consistent with the array data, AQSE treatment led to a dose-dependent decrease in the levels of anti-apoptotic proteins, including Bcl-2, Mcl-1, and Survivin. Conversely, the expression of the pro-apoptotic protein Bax was mildly increased at the highest concentration (60 μg/mL). This shift in the pro-/anti-apoptotic balance was accompanied by the activation of the caspase cascade. AQSE treatment significantly induced the cleavage of caspase-3, as well as the cleavage of PARP1, a hallmark of apoptosis. Taken together, these results suggest that AQSE induces apoptosis in A549 cells by suppressing anti-apoptotic survival signals and activating caspase-3-mediated apoptotic signaling. Given these in vitro findings, the antitumor efficacy of AQSE was evaluated in vivo using a KLN205 syngeneic tumor-bearing mouse model. However, the administration of AQSE, either intraperitoneally (50 mg/kg) or orally (200 mg/kg), did not result in a significant reduction in tumor growth compared to the control group (Figure 4C). No notable changes in body weight were observed during the treatment period. These results suggest that although AQSE induces pronounced apoptosis-associated molecular changes in vitro, these effects alone are insufficient to drive significant tumor growth inhibition in vivo in this model.

### 2.5. AQSE Selectively Modulates MAPK and Survival Signaling Pathways

**Figure 5:**
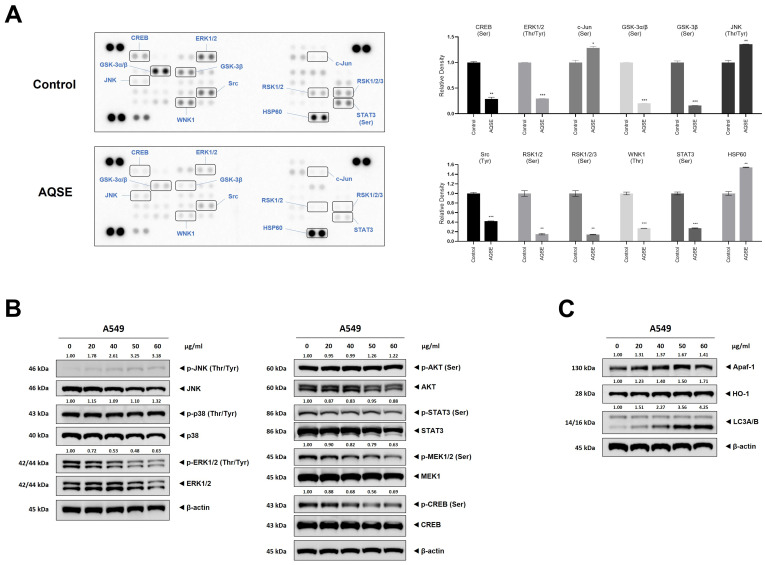
AQSE modulates mitogen-activated protein kinase (MAPK) and survival signaling pathways. (**A**) Phospho-kinase array analysis of A549 cells. Cells were treated with AQSE (50 μg/mL) for 24 h. The images represent the relative phosphorylation levels of various kinases and stress-related proteins. Key changed proteins are highlighted. A quantitative comparison of selected protein levels is shown as the relative density compared to the control. Data are presented as mean ± SD. * *p* < 0.05, ** *p* < 0.01, and *** *p* < 0.001, compared to the untreated control. (**B**) Western blot analysis confirming the regulation of MAPK and survival signaling pathways. (**C**) Western blot analysis of stress- and autophagy-related proteins. A549 cells were treated with the indicated concentrations (0, 20, 40, 50, and 60 μg/mL) of AQSE for 24 h. The expression levels of phosphorylated and total forms of JNK, p38, ERK1/2, AKT, STAT3, MEK1, and CREB, as well as Apaf-1, HO-1, and LC3A/B were analyzed.

To explore the upstream regulatory mechanisms involved in AQSE-induced apoptosis, we investigated the phosphorylation status of kinases that play critical roles in tumor cell survival and stress responses using a phospho-kinase array (Figure 5A). AQSE treatment resulted in a marked reduction in the phosphorylation of several survival- and proliferation-associated kinases, including CREB, ERK1/2, GSK-3, Src, WNK1, RSK, and STAT3. In contrast, the phosphorylation of the stress markers JNK and c-Jun, as well as the expression of HSP60, were increased. Based on these array findings, we further validated the changes in specific signaling pathways by Western blot analysis (Figure 5B). Consistent with the array data, AQSE treatment led to a dose-dependent decrease in the phosphorylation of MEK1/2 and ERK1/2, which are central mediators of cell survival. Furthermore, the phosphorylation of CREB, a downstream target of the ERK pathway, was significantly suppressed. Conversely, the phosphorylation of JNK, a stress-activated protein kinase known to promote apoptotic cell death, was markedly increased following AQSE treatment. Interestingly, the phosphorylation levels of p38 and AKT remained largely unchanged, indicating that AQSE does not inhibit all survival signaling but rather selectively modulates the MEK/ERK and JNK pathways. To further characterize the cellular stress responses associated with AQSE, the expression of stress- and autophagy-related proteins was examined (Figure 5C). AQSE treatment led to a dose-dependent increase in HO-1, Apaf-1, and LC3A/B levels, indicating the induction of stress-associated and autophagy-related cellular responses. Collectively, these results suggest that AQSE creates a cellular environment favorable for cell death by simultaneously inhibiting the survival-promoting MEK/ERK/CREB axis and activating the stress-responsive JNK pathway.

### 2.6. AQSE Induced the Release of DAMPs and Hallmarks of Immunogenic Cell Death

**Figure 6:**
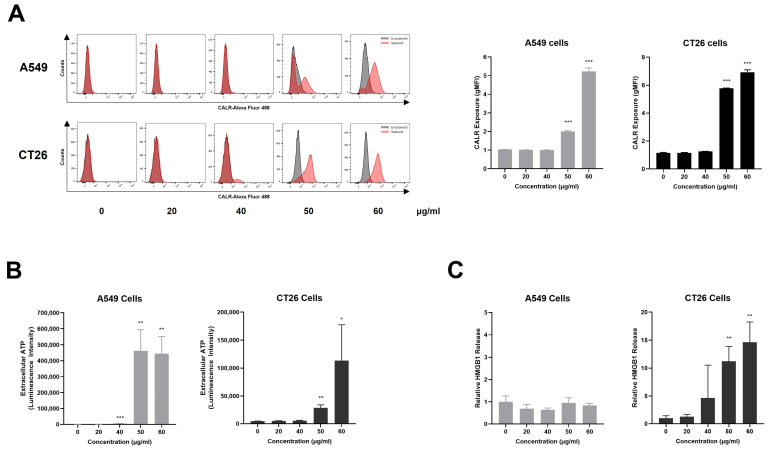
AQSE induces the release of DAMPs; the exposure of CALR, the secretion of ATP, and the release of HMGB1 in cancer cells. A549 and CT26 cells were treated with the indicated concentrations (0, 20, 40, 50, and 60 µg/mL) of AQSE for 24 h. (**A**) Cell surface exposure of CALR. Representative flow cytometry histograms and quantification show increased CALR expression on the surface of A549 and CT26 cells. CALR surface exposure is expressed as the geometric mean fluorescence intensity (gMFI). (**B**) Extracellular ATP levels were quantified in A549 and CT26 cells using a luminescent ATP detection kit. (**C**) HMGB1 concentrations in the culture supernatant were determined by ELISA. Data are presented as mean ± SD. * *p* < 0.05, ** *p* < 0.01, and *** *p* < 0.001, compared to the control group (0 μg/mL).

To determine whether AQSE-induced cell death was accompanied by features of ICD, the expression and release of representative DAMPs, including CALR, ATP, and HMGB1, were examined following AQSE treatment. To assess the surface exposure of CALR, a hallmark event of ICD, flow cytometric analysis was performed. As shown in Figure 6A, AQSE treatment resulted in a marked increase in CALR exposure on the cell surface of both A549 and CT26 cells compared to the untreated controls, indicating the translocation of CALR to the plasma membrane following AQSE treatment. Next, extracellular ATP levels were measured to evaluate AQSE-induced ATP secretion. Luminescence-based ATP assays revealed a significant increase in extracellular ATP levels in both A549 and CT26 cells following AQSE treatment at higher concentrations (50 and 60 μg/mL) (Figure 6B), suggesting the active release of ATP into the extracellular space. In addition, extracellular HMGB1 levels were significantly elevated following AQSE treatment only in CT26 cells (Figure 6C), further supporting the induction of DAMP release upon AQSE exposure. Overall, these data indicate that AQSE induces multiple hallmarks of ICD, suggesting that AQSE-induced cell death is accompanied by immunogenic features.

### 2.7. AQSE-Treated Tumor Cells Acquire Vaccine-like Antitumor Activity In Vivo

**Figure 7:**
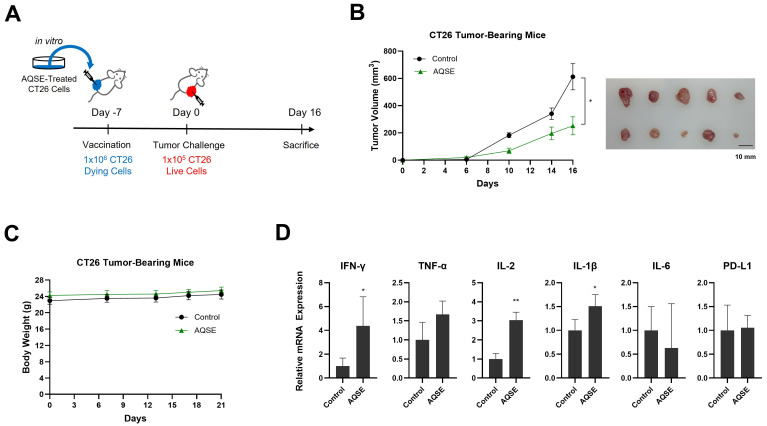
Vaccination of AQSE-treated cells suppresses the growth of the CT26 colorectal tumors. (**A**) Schematic timeline of the vaccination model using CT26 dying and live cell inoculation. CT26 cells were treated with AQSE (50 μg/mL) for 24 h to induce cell death. Dying CT26 cells were inoculated subcutaneously into the left flank of BALB/c mice (*n* = 5 per group). One week later, live CT26 cells were inoculated into the right flank. (**B**) Time courses of CT26 tumor growth and representative images of dissected tumor tissues from control and AQSE-treated groups. Tumor volumes were measured two or three times per week after inoculation. Data are presented as mean ± SEM. Scale bar represents 10 mm. (**C**) Body weight changes in CT26-bearing mice. (**D**) Relative mRNA expression levels of immune-related cytokines (IFN-γ, TNF-α, IL-2, IL-1β, IL-6) and PD-L1 in tumor tissues. The expression levels were analyzed by quantitative Reverse Transcription-PCR. Data are presented as mean ± SD. * *p* < 0.05 and ** *p* < 0.01, compared to the control group.

To investigate the antitumor effects of AQSE-induced ICD in vivo, a vaccination model was established using CT26 cells (Figure 7A). CT26 cells were pretreated with AQSE (50 μg/mL) for 24 h to induce cell death, and the dying cells were inoculated subcutaneously into BALB/c mice. Seven days post-vaccination, live CT26 cells were injected into the contralateral flank to challenge tumor growth. Vaccination with AQSE-treated dying tumor cells resulted in a marked reduction in tumor growth compared to the control group (Figure 7B). Notably, there were no significant changes in body weight between the vaccinated and control groups, indicating that the vaccination did not induce major systemic toxicity (Figure 7C). To determine whether antitumor immune responses were induced in the tumor microenvironment, we analyzed the mRNA expression levels of various cytokines in the tumor tissues (Figure 7D). The vaccinated group exhibited significantly increased expression of IFN-γ, IL-2, and IL-1β, compared to the control group. Conversely, no significant differences were observed in the expression of TNF-α, IL-6, and PD-L1. Overall, these results suggest that AQSE-induced cell death exhibits functional immunogenic properties capable of eliciting a protective antitumor immune response in vivo, which is consistent with the induction of ICD-associated DAMPs observed in vitro.

### 2.8. Fractionation of AQSE Identifies Bioactive Fractions That Suppress NSCLC Cell Proliferation

**Figure 8:**
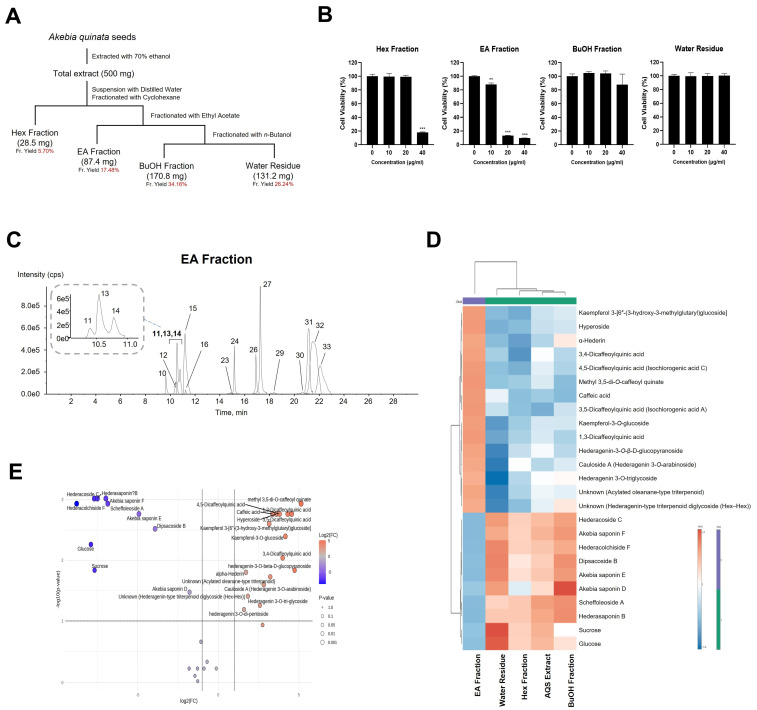
Bioassay-guided fractionation of AQSE and the antiproliferative effects of its fractions. (**A**) Schematic representation of the fractionation procedure. AQSE was suspended in distilled water and sequentially partitioned into cyclohexane (Hex), ethyl acetate (EA), and *n*-butanol (BuOH) fractions. (**B**) The effect of each fraction on the viability of A549 cells. Cells were treated with the indicated concentrations (0, 10, 20, and 40 µg/mL) for 24 h. Cell viability was determined using the CCK-8 assay. Data are presented as mean ± SD (*n* = 3). ** *p* < 0.01 and *** *p* < 0.001, compared to the control group (0 μg/mL). (**C**) Extracted ion chromatogram of the bioactive EA fraction acquired by LC–MS/MS in negative ion mode. (**D**) Heatmap and cluster analysis of the top 25 metabolites. (**E**) Volcano plot identifying differentially abundant metabolites in the EA fraction. The fold change was determined by comparing the peak area of the EA fraction to the median peak area of the AQSE, Hex fraction, BuOH fraction, and water residue. Red and blue circles indicate metabolites with significantly higher and lower abundance in the EA fraction, respectively. Heatmap and volcano plot were generated by MetaboAnalyst 6.0.

To determine whether the antiproliferative effects of AQSE could be attributed to specific chemical fractions, AQSE was fractionated using cyclohexane (Hex), ethyl acetate (EA), and *n*-butanol (BuOH), followed by the water residue (Figure 8A). The resulting fractionation yields were 5.70% (Hex), 17.48% (EA), 34.16% (BuOH), and 26.24% (water residue), with a total recovery of 83.58%. The cytotoxic potential of each fraction was evaluated in A549 cells (Figure 8B). The BuOH fraction and water residue had minimal impact on cell viability across the tested concentration range (0–40 μg/mL). The Hex fraction showed significant toxicity only at the highest concentration (40 µg/mL). In contrast, the EA fraction demonstrated a potent, dose-dependent suppression of cell viability, with significant inhibition observed starting from 10 µg/mL. These results suggest that the bioactive constituents responsible for the anticancer activity of AQSE are primarily concentrated within the EA fraction. Chromatographic profiling and subsequent metabolite identification revealed that the EA fraction is enriched with monodesmosidic saponins containing only C-3 sugar moieties, as well as cinnamic acid and flavonol derivatives. The saponins present within the EA fraction are distinguished by the presence of a particular sugar moiety at the C-3 position and the absence of a sugar moiety at the C-28 position, a feature that distinguishes them from triterpenoid saponins (Figure 8C). To elucidate the chemical basis of this potent cytotoxicity, we performed a comparative metabolomic analysis. The heatmap visualization revealed a distinct metabolic signature for the EA fraction compared to the other fractions (Figure 8D). To statistically identify the specific metabolites contributing to this bioactivity, we conducted a volcano plot analysis (Figure 8E). Notably, distinct classes of bioactive metabolites, including flavonoids (e.g., kaempferol 3-[6″-(3-hydroxy-3-methylglutaryl)glucoside], rutin, and hyperoside), phenylpropanoids (e.g., dicaffeoylquinic acids), and saponins (e.g., hederagenin-3-*O*-β-D-glucopyranoside and α-hederin), exhibited high fold changes and statistical significance in the EA fraction. These findings suggest that the anticancer effect of the EA fraction is likely associated with the combined presence of these specifically enriched flavonoids and saponins. The enrichment of these components in the EA fraction correlates with its antiproliferative effects, indicating that these compounds are the primary contributors to the anticancer and immunogenic properties of AQSE.

## 3. Discussion

Natural products are considered a valuable resource for anticancer drug discovery, as they often exhibit a wide range of pharmacological properties with diverse mechanisms of action [28]. Among these, plant-derived saponins have attracted significant attention due to their potent cytotoxic and immunomodulatory activities [17]. *A. quinata* is a saponin-rich medicinal plant traditionally used for therapeutic purposes, and previous studies have reported its broad pharmacological spectrum, including anti-inflammatory, anti-obesity, and neuroprotective effects [22]. However, limited information is available regarding its anticancer potential. In this study, we investigated the anticancer effects of AQSE, with a particular focus on apoptosis induction and ICD-associated responses in NSCLC cells.

Apoptosis is characterized by two distinct signaling cascades, the intrinsic (mitochondrial) pathway and the extrinsic (death receptor) pathway [29]. Our findings reveal that AQSE primarily induces apoptosis through the intrinsic mitochondrial pathway. Our results demonstrate that AQSE treatment induced the cleavage of caspase-3 and PARP1 and altered the expression of Bcl-2 family proteins, including increased Bax and decreased Bcl-2 and Mcl-1 expressions, indicating the activation of the intrinsic apoptotic pathway. Interestingly, we observed a downregulation of death receptor components, including DR4, DR5, Fas, and FADD. This reduction may reflect a regulatory feedback mechanism, such as ligand-induced receptor internalization and protein degradation occurring during the late stages of cell death [30]. These findings suggest that AQSE-induced apoptosis is predominantly mediated through mitochondrial signaling while secondary regulatory changes in death receptor components may occur during apoptotic progression [31].

The molecular mechanisms underlying AQSE-induced apoptosis appear to be driven by the modulation of the MAPK signaling pathway. MAPKs are central regulators of cellular stress responses and have been implicated in apoptosis, endoplasmic reticulum (ER) stress, and DAMP release [32,33]. MEK-mediated phosphorylation of ERK is a central pro-survival node in NSCLC, and activated ERK propagates mitogenic and anti-apoptotic signaling through downstream kinases such as RSK2 [34]. In turn, RSK2 directly phosphorylates CREB, enabling CREB-dependent transcriptional programs that sustain proliferation, survival, and stress tolerance [35]. Therefore, the inhibition of the MEK-ERK cascade and its downstream transcriptional effector module provides a coherent explanation for the pronounced growth suppression and apoptotic commitment [36]. In this study, AQSE-mediated suppression of MEK phosphorylation was accompanied by reduced ERK1/2 phosphorylation, leading to the inhibition of RSK2 phosphorylation and the subsequent attenuation of CREB activation. This stepwise blockade explains the pronounced growth suppression and apoptotic commitment induced by AQSE. It implies that AQSE silences an ERK-driven survival/transcriptional axis, thereby lowering the apoptotic threshold and limiting adaptive resistance mechanisms.

Interestingly, previous studies have demonstrated that stress-activated MAPKs, particularly JNK and p38, link intracellular stress signaling with immunogenic features of cell death. JNK is a canonical sensor of oxidative, ER, and inflammatory stress, and its activation is frequently linked to mitochondrial apoptotic commitment and cellular stress programs that facilitate ICD-associated danger signaling [37,38]. In line with these observations, AQSE treatment induced the phosphorylation of JNK, providing a mechanistic bridge between cytotoxic stress and immunogenic outputs. Notably, the divergence between enhanced JNK activation and reduced pro-survival ERK activity suggests that AQSE rewires the MAPK circuitry toward stress-dominant, pro-immunogenic cell death trajectories. These results suggest that MAPK modulation contributes to the induction of ICD-associated responses by AQSE, although further studies are required to clarify the precise relationship between specific kinase activities and DAMP release. Furthermore, the upregulation of HO-1, LC3A/B, and Apaf-1 indicates that AQSE triggers integrated cellular stress responses involving oxidative stress, autophagy, and apoptotic signaling, suggesting that AQSE does not merely terminate cell viability but actively reorganizes the cellular state to become more visible to the host immune system.

The induction of effective antitumor immunity remains a major challenge in the treatment of NSCLC [39,40]. Although ICIs have markedly improved clinical outcomes in a subset of patients, the majority of NSCLC cases exhibit limited responsiveness, highlighting the urgent need for alternative or complementary therapeutic strategies [41]. One critical factor contributing to immune resistance is insufficient tumor immunogenicity, which limits the activation of effective antitumor immune responses [42]. In this context, ICD has emerged as a critical mechanism by which dying tumor cells can enhance their immunogenic prolife through the release of DAMPs. Importantly, apoptosis plays a crucial role in determining the immunogenicity of tumor cell death [43]. While apoptosis has traditionally been considered immunologically silent, accumulating evidence demonstrates that apoptosis accompanied by specific cellular stress responses can acquire immunogenic features [4]. In this study, AQSE treatment induced regulated apoptotic cell death rather than nonspecific necrosis. Such a mode of cell death is considered a prerequisite for the induction of ICD, suggesting that AQSE establishes a cellular environment permissive for immunogenic signaling [44]. A hallmark of ICD is the coordinated release of DAMPs, including extracellular ATP and HMGB1, which play essential roles in dendritic cell recruitment, antigen presentation, and T cell priming [45]. In this study, AQSE treatment was associated with a significant increase in extracellular ATP and HMGB1 levels in cancer cells. The observed surface exposure of calreticulin along with the significant release of extracellular ATP and HMGB1 supports the induction of ICD by AQSE.

The immunogenic potential of AQSE-induced cell death was further substantiated by its ability to elicit functional antitumor immunity in vivo. Although our primary focus is on NSCLC, we utilized the CT26 colon carcinoma model for the vaccination assay to provide a proof-of-concept for the immunogenic potential of AQSE. While the KLN205 lung cancer model showed limited sensitivity to direct AQSE administration, the CT26 model is a well-established standard for evaluating antitumor immunological memory and ICD-based vaccination efficacy. AQSE-treated dying CT26 cells generated a protective immune response upon subsequent tumor challenge, indicating that AQSE-induced cell death is capable of activating tumor-specific adaptive immunity rather than merely exerting direct cytotoxic effects. These findings suggest that AQSE-induced ICD has the potential to prime functional antitumor immunity in vivo. While the significant upregulation of IFN-γ and IL-2 strongly implies the activation and recruitment of immune cells, such as cytotoxic T cells, the lack of direct quantitative evidence of immune cell subsets remains a limitation of this study. Further studies are required to characterize the immune cell populations responsible for this response, particularly tumor-infiltrating CD8+ and CD4+ T cells, NK cells, and B cells. Nevertheless, this observation provides a mechanistic rationale that the ICD hallmarks observed in vitro in NSCLC cells can indeed translate into functional antitumor immunity in vivo, warranting future investigations using optimized delivery systems or combination strategies in lung cancer models. Notably, despite the potent in vitro pro-apoptotic effects, AQSE treatment did not suppress the tumor growth in the KLN205 syngeneic mouse model. The discrepancy between the potent in vitro cytotoxicity and the limited in vivo therapeutic efficacy observed during direct systemic administration highlights critical pharmacokinetic challenges. Factors such as poor aqueous solubility and rapid metabolism may prevent the bioactive compounds from reaching effective concentrations within the tumor microenvironment. These limitations suggest that the primary value of AQSE in its current form may lie in its ability to prime the immune system through ICD rather than exerting direct systemic toxicity. Furthermore, while our vaccination data demonstrate the establishment of antitumor immunity, future studies employing detailed immunophenotyping analyses such as flow cytometry, will be necessary to precisely quantify tumor-infiltrating immune cell populations, including CD8+ T cells within the tumor microenvironment.

The immunogenic effects observed following AQSE treatment are consistent with the known biological properties of saponins, which are reported to be the major bioactive constituents of *A. quinata*. Saponins possess amphipathic structures that enable interactions with cellular membranes and have been widely recognized for their adjuvant-like activity in vaccine formulations [46]. Given that AQSE is a saponin-rich extract, its ability to induce ICD-associated DAMP release is likely attributable to these intrinsic immunomodulatory characteristics. To validate this and identify the specific active constituents responsible for these effects, we extended our investigation using solvent fractionation. While crude extracts often exhibit broad biological activities due to the synergistic action of multiple compounds, our fractionation analysis enabled the identification of specific fractions that are primarily responsible for the observed apoptotic and ICD-associated effects. Notably, the antiproliferative effect of the EA fraction strongly suggests that the anticancer potential of AQSE is not driven by the saponins abundant in the polar fractions, but rather by a distinct group of bioactive metabolites preferentially enriched in the EA fraction. Our metabolomic profiling revealed that the bioactive EA fraction was notably enriched with sapogenins (aglycones) and less-glycosylated saponins. Specifically, hydrophobic compounds such as hederagenin and its derivatives (α-hederin and cauloside A) were preferentially partitioned into the EA layer. These compounds often possess fewer sugar moieties or distinct structural features that render them relatively less polar. In addition, our analysis identified flavonoids and phenolic acids as the key contributors to this potency. The significant enrichment of these compounds in the bioactive EA fraction implies that they may possess the membrane permeability or specific binding affinities required to trigger intracellular stress signaling. Furthermore, the co-enrichment of flavonoids and saponins in the EA fraction suggests a potential synergistic mechanism, where saponins may facilitate the cellular uptake of flavonoids or collectively amplify ROS generation and ER stress, thereby potentiating the induction of ICD [47]. This approach highlights the importance of dissecting complex natural extracts to delineate their functionally relevant bioactive components. Collectively, the present study provides critical mechanistic insight into the molecular basis of the anticancer activity of AQSE and establishes a clear rationale for prioritizing these specific metabolites for future bioassay-guided isolation and drug development.

## 4. Materials and Methods

### 4.1. Materials and Reagents

The dried seeds of *A. quinata* were provided by Dr. Changjin Kim from Woori Madi Hospital (Jeonju, Republic of Korea) and morphologically authenticated by Dr. Goya Choi, an herbalist at the Korea Institute of Oriental Medicine (Naju, Republic of Korea). A voucher specimen (AQSE-2021) was deposited at the KM Science Research Division, Korea Institute of Oriental Medicine (Daejeon, Republic of Korea). Cell Counting Kit-8 (CCK-8) was purchased from Dojindo (Kumamoto, Japan). Dulbecco’s phosphate-buffered saline, Roswell Park Memorial Institute (RPMI) 1640 medium, and fetal bovine serum (FBS) were purchased from Welgene (Daegu, Republic of Korea). Trypsin-EDTA solution and penicillin/streptomycin were obtained from Gibco (Grand Island, NY, USA). Antibodies against cleaved PARP, cleaved caspase-3, Survivin, Bcl-2, Bax, Mcl-1, Apaf-1, HO-1, LC3A/B, p-JNK (Thr183/Tyr185), JNK, p-p38 (Thr180/Tyr182), p38, p-ERK1/2 (Thr202/Tyr204), ERK1/2, p-AKT (Ser473), AKT, p-STAT3 (Ser727), STAT3, p-MEK1/2 (Ser217/221), MEK1, p-CREB (Ser133), CREB, and β-actin, were purchased from Cell Signaling Technology (Danvers, MA, USA).

### 4.2. Preparation of AQSE

Dried seeds of *A. quinata* were extracted with 70% ethanol using an ultrasonic extraction method [48]. Briefly, 300 g of dried and ground *A. quinata* seeds were extracted with 500 mL of 70% ethanol by ultrasonication for 1 h at room temperature. After filtration, the extraction procedure was repeated three times. The combined extracts were concentrated under reduced pressure and lyophilized to obtain the AQSE as a powder (21.6 g; 7.2% yield). The extract was stored at −20 °C until further analysis.

### 4.3. LC–MS/MS Analysis

A Thermo Scientific Vanquish UHPLC system (Thermo Fisher Scientific, Sunnyvale, CA, USA) equipped with an Acquity UPLC HSS T3 column (2.1 mm × 100 mm, 2.7 μm, Waters, Milford, MA, USA) coupled to a Triple TOF5600^+^ mass spectrometer system (QTOF MS/MS, SCIEX, Foster City, CA, USA) was used. The QTOF MS was equipped with an electrospray ionization (ESI) source operating in negative ion mode and used to complete the high-resolution experiment.

### 4.4. Fractionation of AQSE

AQSE was subjected to solvent fractionation to obtain active fractions. Briefly, AQSE (500 mg) was suspended in 150 mL of distilled water and fractionated through sequential liquid–liquid partitioning using 150 mL each of Hex, EA, BuOH in order of increasing polarity. The resulting organic fractions and the residual aqueous phase were concentrated under reduced pressure using a rotary evaporator and subsequently freeze-dried.

### 4.5. Cell Culture

A549 and H460 human NSCLC cells and CT26 murine colorectal cancer cells were purchased from the Korean Cell Line Bank (Seoul, Republic of Korea). KLN205 murine lung cancer cells were obtained from the American Type Culture Collection (Rockville, MD, USA). A549, H460, and CT26 cells were maintained in RPMI 1640 medium and KLN205 cells were cultured in EMEM, supplemented with 10% FBS and 1% penicillin–streptomycin. Cells were incubated at 37 °C in a humidified 5% CO_2_ atmosphere.

### 4.6. Cell Proliferation Assay

Cell viability was determined using the CCK-8 assay. Briefly, A549, H460, and KLN205 cells were treated with AQSE (0–80 μg/mL) for 24 h. Then, 10 µL of CCK-8 reagent was added to each well and incubated for 1 h. The absorbance of the CCK-8 reagent was measured at 450 nm using a SpectraMax i3 microplate reader (Molecular Devices, San Jose, CA, USA).

### 4.7. Flow Cytometry Analysis 

A549, H460 and CT26 cells were seeded at a density of 5 × 10^5^ cells/well and incubated overnight. Cells were then treated with the indicated concentrations of AQSE for 24 h and subsequently harvested. Apoptotic cell death was assessed by staining with Annexin V-FITC and 7-AAD. For cell cycle analysis, cells were stained with propidium iodide to determine the sub-G1 population, which reflects apoptotic DNA fragmentation. For the analysis of cell surface CALR exposure, a separate aliquot of cells was incubated with Alexa Fluor 488 conjugated anti-calreticulin antibodies. The stained cells were acquired and analyzed using a BD LSRFortessa™ X-20 flow cytometer (BD Biosciences, San Diego, CA, USA) and FlowJo 10.0 software (BD Biosciences).

### 4.8. Proteome Array Analysis

A549 and H460 cells were seeded onto 6-well plates at a density of 5 × 10^5^ cells/well and incubated overnight. The cells were treated with 50 µg/mL of AQSE for 24 h. After incubation, changes in the expression of apoptosis-related proteins were assessed using the Human Apoptosis Array kit (R&D Systems, Minneapolis, MN, USA). The expression levels of phospho-kinases were examined using the Proteome Phospho-Kinase Array kit (R&D Systems). The blots were developed using Clarity ECL Western Blotting Substrates (Bio-Rad, Hercules, CA, USA) and visualized using an ImageQuant LAS 4000 mini system (GE Healthcare, Chicago, IL, USA). The densitometric analysis of the array spots was performed using ImageJ version 1.54 software (National Institutes of Health, Bethesda, MD, USA).

### 4.9. Western Blot Analysis

5 × 10^5^ cells/well were seeded onto 6-well plates overnight. The cells were treated with various concentrations of AQSE for 24 h and lysed using Cell Lysis Buffer II (Invitrogen, Carlsbad, CA, USA), supplemented with phenylmethylsulfonyl fluoride and Phosphatase Inhibitor Cocktail (Thermo Fisher Scientific). Protein concentration was quantified using the Qubit™ Protein Assay Kit (Invitrogen). Protein extracts were mixed with 4× LDS sample buffer and 10× reducing agent, followed by heating at 75 °C for 10 min. Samples were separated on 10–12% Bis-Tris gels using MOPS SDS running buffer and transferred onto 0.45 µm nitrocellulose membranes using the SureLock Tandem Transfer System (Thermo Fisher Scientific). Membranes were blocked with EzBlock Chemi (ATTO, Tokyo, Japan) at room temperature and incubated with primary antibodies at 4 °C overnight, and incubated with secondary antibodies conjugated with horseradish peroxidase for 2 h. The proteins were detected using SuperSignal West Femto Maximum Sensitivity Substrate (Thermo Fisher Scientific) and visualized with an ImageQuant LAS 4000 mini system (GE Healthcare, Chicago, IL, USA).

### 4.10. Animal Experiments

Specific pathogen-free four-week-old male DBA/2 and BALB/c mice were purchased from Saeron Bio (Uiwang, Republic of Korea) and acclimated for one week before experimental use. Mice were housed under controlled conditions (temperature: 23 °C; humidity: 50%; 12h light/dark cycle). Animals were fed a standard chow diet (Purina Co., Seoul, Republic of Korea) and provided drinking water ad libitum. All experimental procedures were approved by the Animal Care and Use Committee of the Korea Institute of Oriental Medicine (Approval number: 25-009). To establish the KLN205 cell tumor-bearing mice, 1 × 10^5^ KLN205 cells were subcutaneously inoculated into the right flank of DBA/2 mice. After tumor challenge, DBA/2 mice were administered AQSE intraperitoneally (50 mg/kg) or orally (200 mg/kg) every other day. For the vaccination model, CT26 cells were treated with AQSE (50 μg/mL) for 24 h to induce ICD. The resulting dying CT26 cells (1 × 10^6^) were inoculated subcutaneously into the left flank of BALB/c mice. One week after immunization, live CT26 cells (1 × 10^5^) were subcutaneously injected into the right flank. Tumor volume was measured using calipers and calculated using the following formula: length × width^2^ × 1/2. Body weight and tumor size were monitored during the experiments.

### 4.11. Measurement of Extracellular ATP and HMGB1

The extracellular ATP levels were measured using the RealTime-Glo Extracellular ATP Assay kit (Promega, Madison, WI, USA) according to manufacturer instructions. Briefly, cells at densities of 1 × 10^4^ cells/well (A549 and CT26) were cultured in opaque-walled 96 well plates in L15 medium with 10% FBS. After AQSE treatment, RealTime-Glo Extracellular ATP Assay Reagent was added to each well and Mitoxantrone was used as a positive control. The luminescence intensity was measured for 24 h. The amount of HMGB1 protein in the supernatant was determined using human HMGB1-specific ELISA kits (Cusabio, Houston, TX, USA) according to the manufacturer’s instructions. ELISA was employed to measure the HMGB1 concentration in the supernatants and cytoplasmic lysates of CT26 cells.

### 4.12. Quantitative Reverse Transcription (qRT)-PCR

Total RNA was extracted using the RNeasy Plus Mini Kit (Qiagen, Valencia, CA, USA). RNA concentration and purity were assessed using Nanodrop spectrophotometer (Thermo Scientific). 1 μg of total RNA was reverse-transcribed into cDNA using the iScript™ Advanced cDNA Synthesis kit (Bio-Rad). qRT-PCR was performed using SsoAdvanced™ SYBR Green Supermix (Bio-Rad) on a Bio-Rad CFX Connect Real-Time PCR system. Amplification conditions consisted of an initial denaturation at 95 °C for 2 min, followed by 40 cycles of amplification at 95 °C for 5 s and 60 °C for 30 s. Gene expression levels were calculated using the 2^−ΔΔCt^ method and normalized by the housekeeping gene ACTB. The specific primer sequences used in this study were as follows: mouse IFN-γ (forward: 5′-GAG CCA GAT TAT CTC TTT CTA CCT-3′; reverse: 5′-GTT GTT GAC CTC AAA CTT GGC-3′), IL-1β (forward: 5′-TGG ACC TTC CAG GAT GAG GAC A-3′; reverse: 5′-GTT CAT CTC GGA GCC TGT AGT G-3′), IL-2 (forward: 5′-GCG GCA TGT TCT GGA TTT GAC TC-3′; reverse: 5′-CCA CCA CAG TTG CTG ACT CAT C-3′), IL-6 (forward: 5′-TAC CAC TTC ACA AGT CGG AGG C-3′; reverse: 5′-CTG CAA GTG CAT CAT CGT TGT TC-3′), TNF-α (forward: 5′-CCA CGT CGT AGC AAA CCA C-3′; reverse: 5′-TTG TCC CTT GAA GAG AAC CTG-3′), PD-L1 (forward: 5′-TGC TGC ATA ATC AGC TAC GG-3′; reverse: 5′-GCT GGT CAC ATT GAG AAG CA-3′), and ACTB (forward: 5′-CCT TCT TGG GTA TGG AAT CCT G-3′; reverse: 5′-CAA TGC CTG GGT ACA TGG TG-3′).

### 4.13. Statistical Analysis

All statistical analyses were performed using GraphPad Prism 10 software (San Diego, CA, USA). Data are presented as mean ± standard deviation (SD), except for tumor growth, which are expressed as mean ± standard error of the mean (SEM). Statistical significance was determined using a two-tailed Student’s *t*-test or one-way ANOVA followed by Dunnett’s post hoc test. For mRNA expression analysis, a one-tailed *t*-test was used. *p* < 0.05 was considered statistically significant.

## 5. Conclusions

The present study demonstrates that AQSE exerts potent anticancer effects against NSCLC cells by inducing regulated apoptotic cell death and the release of ICD-associated DAMPs. Mechanistically, these effects are driven by the coordinated modulation of MAPK signaling pathways, in which the suppression of pro-survival kinase activity and the activation of stress-associated signaling converge to promote immunogenic cell death. This multilayered mechanism highlights the unique advantage of AQSE as a natural product-based agent capable of not only exerting direct cytotoxicity but also potentially overcoming immune resistance. Consequently, our findings establish a strong rationale for further investigating AQSE as a promising immunogenic anticancer candidate for the treatment of NSCLC.

## Figures and Tables

**Table 1:** Qualitative analysis and metabolite identification of chemical components in AQSE by UHPLC–QTOF MS/MS-based chemical profile analysis.

No.	Name	Formula	Adduct	Found at Mass (Da)	Error (ppm)
1	Glucose	C_6_H_12_O_6_	[M–H]^–^	179.0563	1.0
2	Sucrose	C_12_H_22_O_11_	[M–H]^–^	341.1086	−1.1
3	Citric acid	C_6_H_8_O_7_	[M–H]^–^	191.0202	2.5
4	Neochlorogenic acid	C_16_H_18_O_9_	[M–H]^–^	353.0875	−0.9
5	Chlorogenic acid	C_16_H_18_O_9_	[M–H]^–^	353.0872	−1.7
6	Caffeic acid	C_9_H_8_O_4_	[M–H]^–^	179.0351	0.5
7	Cryptochlorogenic acid	C_16_H_18_O_9_	[M–H]^–^	353.0872	−1.7
8	Quercetin-3-*O*-arabinoglucoside	C_26_H_28_O_16_	[M–H]^–^	595.1296	2.3
9	Rutin	C_27_H_30_O_16_	[M–H]^–^	609.1448	−1.2
10	Hyperoside	C_21_H_20_O_12_	[M–H]^–^	463.0872	−2.1
11	3,4-Dicaffeoylquinic acid	C_25_H_24_O_12_	[M–H]^–^	515.1191	−0.8
12	Kaempferol-3-*O*-glucoside	C_21_H_20_O_11_	[M–H]^–^	447.0915	−1.8
13	1,3-Dicaffeoylquinic acid	C_25_H_24_O_12_	[M–H]^–^	515.1190	−0.9
14	3,5-Dicaffeoylquinic acid (Isochlorogenic acid A)	C_25_H_24_O_12_	[M–H]^–^	515.1191	−0.9
15	4,5-Dicaffeoylquinic acid (Isochlorogenic acid C)	C_25_H_24_O_12_	[M–H]^–^	515.1192	−0.7
16	Kaempferol 3-[6″-(3-hydroxy-3-methylglutaryl)glucoside]	C_27_H_28_O_15_	[M–H]^–^	591.1340	−1.7
17	Methyl 3,5-di-*O*-caffeoyl quinate	C_26_H_26_O_12_	[M–H]^–^	529.1330	−2.1
18	Hederacolchiside F	C_65_H_106_O_31_	[M+FA–H]^–^	1427.6670	−0.9
19	Scheffoleoside A	C_48_H_78_O_19_	[M+FA–H]^–^	1003.5093	−1.5
20	Hederacoside C	C_59_H_96_O_26_	[M+FA–H]^–^	1265.6154	−0.4
21	Akebia saponin F	C_53_H_86_O_23_	[M+FA–H]^–^	1135.5525	−0.5
22	Akebia saponin E	C_52_H_84_O_22_	[M+FA–H]^–^	1105.5420	−0.5
23	Dipsacoside B	C_53_H_86_O_22_	[M+FA–H]^–^	1119.5580	−0.2
24	Akebia saponin D	C_47_H_76_O_18_	[M+FA–H]^–^	973.4998	−0.4
25	Hederasaponin B	C_59_H_96_O_25_	[M+FA–H]^–^	1249.6208	−0.3
26	Unknown (Hederagenin-type triterpenoid diglycoside (Hex–Hex))	C_47_H_76_O_17_	[M+FA–H]^–^	957.5051	0.3
27	Unknown (Acylated oleanane-type triterpenoid)	C_48_H_62_O_8_	[M+FA–H]^–^	811.4452	2.4
28	Hederagenin 3-*O*-triglycoside	C_47_H_74_O_17_	[M+FA–H]^–^	955.4897	2.1
29	Hederagenin-3-*O*-β-D-glucopyranoside	C_36_H_58_O_9_	[M+FA–H]^–^	679.4052	2.0
30	Hederagenin	C_30_H_48_O_4_	[M–H]^–^	471.3466	−2.0
31	Hederagenin 3-*O*-dipentoside	C_40_H_64_O_12_	[M+FA–H]^–^	781.4371	0.3
32	α-Hederin	C_41_H_66_O_12_	[M+FA–H]^–^	795.4522	−0.5
33	Cauloside A (Hederagenin 3-*O*-arabinoside)	C_35_H_56_O_8_	[M+FA–H]^–^	649.3961	2.3
34	Linoleic acid	C_18_H_32_O_2_	[M–H]^–^	279.2329	−0.4
35	Palmitic acid	C_16_H_32_O_2_	[M–H]^–^	281.2483	−0.9

## Data Availability

The original data presented in this research are included in the article. Further inquiries can be directed to the corresponding author.
